# Indirect Effects of the Coping Power Intervention on Latent Suicidal Thoughts and Behaviors: an Integrative Data Analysis

**DOI:** 10.1007/s11121-026-01918-y

**Published:** 2026-05-11

**Authors:** Antonio A. Morgan-López, Stephen G. West, Lissette M. Saavedra, Nisha C. Gottfredson O’Shea, Heather L. McDaniel, Alexandra T. Tonigan, Alexa C. Budavari, Ali Ünlü, Nicole P. Powell, Lixin Qu, Anna C. Yaros, Catherine P. Bradshaw, John E. Lochman

**Affiliations:** 1 RTI International, Center for Behavioral Health and WellBeing, Research Triangle Park, NC USA; 2 Department of Psychology, Arizona State University, Tempe, AZ USA; 3 Department of Methods and Evaluation, Freie Universität Berlin, Berlin, Germany; 4 Department of Psychology, University of South Carolina, Columbia, SC USA; 5 Institute for Social Research, University of New Mexico, Albuquerque, NM USA; 6 School of Education and Human Development, University of Virginia, Charlottesville, VA USA; 7 Center for Youth Development and Intervention, University of Alabama, Tuscaloosa, AL USA; 8 Bloomberg School of Public Health, Johns Hopkins University, Baltimore, MD USA

**Keywords:** Suicide, Mediation analysis, IPD meta-analysis

## Abstract

There has been growing interest in preventive intervention “crossover” effects on suicidal thoughts and behaviors (STBs), in which targeting early risk factors may mitigate distal risk for STBs without STBs having been the targeted outcomes of the primary study. The present study extends an 11-study integrative data analysis of the Coping Power (CP) intervention (*N* = 3182) to assess indirect effects of different forms of CP on teacher- and parent-reported STBs as transmitted through different subdimensions of internalizing and externalizing problems. Compared to school-as-usual, all forms of CP (Standard/Group CP, Individual CP, CP with Mindfulness, Internet-Enhanced CP) led to reductions in parent- and/or teacher-reported youth STBs. Subgroup analyses suggested that boys benefitted from Individual CP and CP with Mindfulness mediated by reductions in aggressive behavior, whereas boys in Standard CP saw reductions in STBs mediated by reductions in conduct problems. Girls saw reductions in STBs in CP with Mindfulness mediated by reductions in anxiety. Some inferences made for individual $\widehat{\alpha }$ and $\widehat{\beta }$ paths and mediation effects differed when using standard parametric approaches for inference versus bias-corrected percentile bootstrapping. These differences highlight cautions regarding statistical inference for prevention researchers who study highly skewed zero-inflated latent variables such as STBs. Findings are discussed in light of (a) earlier etiological research on biological sex-specificity in the pathways to early risk for suicide and (b) how variation in program components of CP and its adaptations may reduce STB risk across different populations, age groups, and modes of program delivery.

**Supplementary Information:**

The online version contains supplementary material available at 10.1007/s11121-026-01918-y.

Death by suicide is one of the most devastating of all human behaviors and is consistently one of the leading causes of death worldwide. Over 800,000 individuals in the USA die by suicide annually, with a person dying by their own hands every 40 s (Nock et al., 2016; Stone et al., 2018). Suicide is the second leading cause of death among individuals aged 10–34 in the USA, with 17% of high school-aged youth seriously considering a suicide attempt and 8% attempting suicide (CDC, 2016). Although there are several behavioral and pharmacological treatments available for addressing psychiatric disorders that are known precursor conditions to suicidality (e.g., depression, anxiety), the overall mortality rate for suicide has continued to increase. Much of this disconnect between treatment availability and the rise in suicide rates has been attributable to low levels of recognition of the treatment needs for serious mental illnesses (SMIs) in community settings, low rates of uptake and use of evidence-based treatments, and difficulties in recognizing imminent risk for suicide even among those seeking treatment for SMIs (Brent, 2016).

In part because it has been difficult to reduce suicide rates by treating SMIs, there has been increased interest in understanding the optimal mechanisms for mitigating the early risk conditions that lead to later suicidal thoughts and behaviors (STBs), attempts, and completion (Ayer et al., 2023; Reider & Sims, 2016). Collectively, investigators have made three key arguments: (1) the most critical period for the onset, and thus the prevention, of suicidal thoughts and behaviors is during childhood and early adolescence; (2) targeting lower-risk conditions that serve as intermediate endpoints of suicide risk (i.e., critical outcomes in and of themselves) such as youth onset SMIs and substance use can mitigate risk for suicide later in life; and (3) the largest impact on suicide risk would come from preventive interventions that target multiple risk factors and/or intermediate endpoints simultaneously (King et al., 2018; Wyman, 2014). Targeting at least one disorder with an intervention that leads to positive effects on other outcomes that were not originally targeted (i.e., “crossover” effects; Kendall & Kessler, 2002) is a particularly promising approach for preventing suicidal behaviors because youth onset SMI is twice as likely as adult onset SMI to lead to a suicide attempt (Brent, 2016).

Both theory and empirical findings from the developmental psychopathology literature suggest a multitude of potential intervention targets for suicide-risk reduction such as (1) building coping skills for managing stress, (2) increasing child-parent (and general youth-adult) bonding, and (3) enhancing emotion regulation for both children and parents (Brent, 2016; Lochman & Dodge, 1994). Although many of the preventive interventions that addressed these and other intervention targets were not designed explicitly for the reduction of suicide behaviors, some of these interventions have shown downstream reductions in suicide-specific risk markers (Bai et al., 2025; Connell et al., 2016; Sandler et al., 2016; Vidot et al., 2016; see also Wilcox et al., 2008). As part of work featured in a 2016 Special Issue on crossover effects in the journal *Suicide and Life*-*Threatening Behavior* (Reider & Sims, 2016), three specific calls were made within the field: (a) greater attention to the “pool[ing] of data across prevention studies” (Reider & Sims, 2016, p. S6; see also Pearson & Sims, 2023) to increase statistical power in detecting crossover effects, important given the low base rate of STBs; (b) the examination of STB crossover effects from interventions that targeted externalizing as opposed to those that had focused on internalizing problems (Brent, 2016); and (c) examination of biological sex-specificity in differential pathways to the mitigation of STB (Morgan-López et al., 2022).

Youth STBs are uncommon, making the detection of intervention effects on STBs challenging (Ayer et al., 2023). A key recommendation from the National Institutes of Health (NIH) Office of Disease Prevention’s (ODP), *Pathways 2 Prevention* (P2P) meeting in 2016 (Goldstein & Avenevoli, 2018; Wilcox et al., 2016) was to promote the integration of multiple studies of the same intervention to achieve adequate statistical power to detect crossover intervention effects on STBs; increases in statistical power are one of the key benefits of integrative data analysis (IDA; Curran et al., 2008, 2014). Results from initial IDA studies of crossover effects on STBs have been promising (see Pearson & Sims, 2023). For example, an IDA study that combined three different interventions (i.e., Good Behavior Game (GBG; Kellam et al., 2008, 2014), Fast Track (Goulter et al., 2019), and Linking the Interests of Families and Teachers Study [LIFT; Eddy et al., 2000, 2003]) examined (a) whether these interventions targeting youth problem behaviors mitigated risk for suicidal ideation and suicide attempts from late adolescence through age 32, and (b) the extent to which intervention effects were mediated by reductions in psychosis (Musci et al., 2023). An IDA of three Family Check-Up (FCU; Stormshak & Dishion, 2009; Stormshak et al., 2011) randomized controlled trials, one implemented in early childhood and two implemented in 6th grade, showed that FCU reduced latent STB risk through young adulthood (Connell et al., 2023), although the FCU versus control differences waned over time. Similarly, an examination of multiple forms of the Coping Power intervention (CP; Boxmeyer et al., 2021; Lochman & Wells, 2002; Lochman et al., 2015, 2017, 2019) in an 11-study IDA (Morgan-López et al., 2022) showed that both teacher- and parent-reported STBs were reduced by CP compared to the school-as-usual control group when CP was delivered over the internet or with a mindfulness component (McDaniel et al., 2025); such reductions relative to the school as usual control group were not apparent for Standard CP nor Individual CP.

Despite these recent IDA advances, there remain significant gaps. First, few studies have examined mediation effects with regard to the factors that were the original intervention targets that were intermediate in the causal sequence that leads to STB risk. In a notable example, Musci et al. showed that elevated-but-decreasing early *psychosis* trajectories, that were not the original intervention targets (Ialongo et al., 1999), were related to later young adult risk for suicidal ideation and attempts. Yet, the GBG/FT/LIFT interventions did not impact psychosis trajectories compared to control conditions. Seidman et al. (2025) found indirect effects for the FCU intervention on STBs operating through self-regulation within the above-mentioned IDA (Connell et al., 2023), that did not reach the level of statistical significance. Second, MacKinnon et al. (2000) noted that the lack of an effect on an outcome when examining overall—or *total*—effects does not necessarily imply the absence of mediation. It is possible to have a null total effect due to “inconsistent mediation,” in which the mediated (indirect) effect and the direct effect are of equal magnitude but have opposite signs: the direct and indirect effects cancel yielding a null total effect (MacKinnon et al., 2000; see MacKinnon, 2000 and Saavedra et al., 2023 for applied examples). MacKinnon and colleagues’ observation is important in relation to the findings in McDaniel et al. (2025) which found that neither Standard nor CP and Individual CP showed total effects on STBs, even though they have shown total effects for on other outcomes (Lochman & Wells, 2002, 2004; Lochman et al., 2015, 2019). Thus, it is possible that mediation effects on STBs may exist for these two interventions. Third, in the literature on intervention crossover effects on STBs, both in single-studies (Connell et al., 2016, 2023; Sandler et al., 2016; Vidot et al., 2016; Wilcox et al., 2008) and IDAs (Connell et al., 2023; Musci et al., 2023), there has been a strong emphasis on examining STB crossover effects from *internalizing*-focused interventions, whereas parallel examination of potential crossover effects of *externalizing*-focused interventions have rarely been undertaken (Brent, 2016; McDaniel et al., 2025; Morgan-López et al., 2022).

## The Present Study

The present study extends prior analyses (McDaniel et al., 2025) of the 11-study IDA of Coping Power, by assessing indirect effects of different forms of CP on teacher- and parent-reported STBs through different subdimensions of internalizing (i.e., anxiety, depression, somatization) and externalizing problems (i.e., conduct problems, aggression, hyperactivity). Coping Power was developed and tested as an indicated preventive intervention to be delivered in the late elementary school years in school-based small groups for at-risk children with aggressive behavior problems. The full standard Coping Power program has 34 child group sessions, and 16 parent group sessions. The CP child component focuses on goal-setting, emotion regulation, perspective-taking, problem-solving, and developing less deviant peer relations. The CP parent component focuses on enhancing positive parenting, providing consistent antecedents and consequences for children’s behavior, reducing harsh and inconsistent parenting, enhancing family cohesion, and increasing parents’ own stress management. Following efficacy studies that established the evidence-base for the program (Lochman & Wells, 2004), CP served as a foundation for testing developmental adaptations (Early Adolescent CP), for testing different formats and lengths of the program (abbreviated standard Coping Power; Individual CP), and for testing optimized versions of the program (CP with Mindfulness; Internet-enhanced CP), but all of these adaptations retained the same focus on the underlying mechanisms targeted by CP.

Previous analyses of STB outcomes in this integrated dataset have found that different forms of delivery of CP (Standard CP, Individual CP, CP with Mindfulness, Internet-enhanced CP) have different intervention effects compared to school-as-usual. It is possible that these different forms of CP may also operate through different intermediate endpoints (i.e., mediators that are not intervention mechanisms per se) to affect STB outcomes. Although many other non-STB studies treat both internalizing and externalizing as unidimensional constructs (e.g., Michael et al., 2016), recent work has shown that many preventive interventions exert differential effects on subdimensions of internalizing and externalizing. In particular, CP may have different direct versus indirect impacts on psychiatric outcomes depending on the particular internalizing/externalizing subdimension (McDaniel et al., 2023b; Saavedra et al., 2025).

In addition to disentangling indirect and direct effects of CP on STBs, there is reason to examine both age and biological sex-specificity in STB outcomes. For example, McDaniel et al. (2025) showed that different forms of CP may be more effective for youth when delivered in late elementary school (Lochman et al., 2019) versus middle or high school (Bradshaw et al., 2025). This suggests additional value of exploring variation across developmental stages in direct and indirect effects of CP on STBs. There is also a substantial literature showing biological sex differences in pathways to suicidal thoughts, behaviors, attempts, and completion, such that boys are more likely to have had externalizing problems, whereas girls are more likely to have had internalizing problems prior to a suicide attempt (Brent et al., 1999; Rhodes et al., 2014). Although other studies have examined internalizing and externalizing as mediators of prevention “crossover” effects on STBs (e.g., Wilcox et al., 2008), few studies have examined biological sex-specificity in these intervention pathways, which would also serve as a test of whether these etiological pathways to suicidality can be altered by intervention. The primary hypothesis of the overall research project, that the adaptations of the CP intervention would show greater impacts on STBs compared to school-as-usual (and SCP), was already examined in McDaniel et al. (2025); the primary hypotheses tested in this analysis are that (a) CP and its adaptations will show indirect effects on STBs overall (compared to school-as-usual) and (b) the adaptations of CP will reduce STBs through reductions in externalizing for boys but through reductions in internalizing for girls (see pre-registered hypotheses in Morgan-López et al., 2022). Developmental differences in the indirect effects of CP were examined in an exploratory fashion.

## Method

### Participants

Data come from 11 RCTs of CP conducted in schools (9 elementary, 1 middle, 1 high) across three states (Alabama, Maryland, North Carolina). Youth were screened into each trial based on teacher-reported (and sometimes parent-reported) screener scores indicating elevated levels of aggressive behavior. The sample was made up of 36.4% female youth and 77.3% Black youth across the 11 RCTs. Demographic information is reported in Table 1, with additional detail regarding study design not otherwise covered below are presented in McDaniel et al. (2025) and Morgan-López et al. (2022).

**Table 1:** Descriptives at baseline

Variable	SAU		SCP		ICP		MCP		IECP		Tier 1		Raw *p*-value	MLM *p*-value
	*M*	*SD*	*M*	*SD*	*M*	*SD*	*M*	*SD*	*M*	*SD*	*M*	*SD*		
Grade level	6.13	2.02	5.86	1.97	4	0	4	0	4	0	4	0	<.001	0.67
% Female	36.3%		35.5%		37.1%		36.5%		40.8%		36.4%		0.75	0.93
% Black	76.7%		77.0%		72.3%		90.0%		89.8%		86.6%		<.001	0.84
Family income													0.09	0.47
None	5.4%		6.1%		5.2%		9.8%		4.1%		4.5%			
< $15,000	28.3%		28.3%		24.8%		23.5%		32.7%		40.7%			
$15,000–$29,999	28.9%		26.8%		30.5%		25.5%		30.6%		22.8%			
$30,000–$49,999	24.2%		25.5%		26.2%		33.3%		16.3%		18.7%			
> $50,000	13.2%		13.4%		13.3%		7.8%		16.3%		13.4%			
Parent-reported STB score	− 0.27	0.33	− 0.03	0.65	0.01	0.68	− 0.06	0.67	− 0.17	0.46	− 0.12	0.56	0.15	0.37
Teacher-reported STB score	− 0.04	0.48	− 0.16	0.39	− 0.16	0.42	− 0.26	0.19	− 0.23	0.39	− 0.2	0.43	0.22	0.68
Teacher-reported conduct problems score	− 0.05	0.93	0.07	0.94	0.44	0.81	− 0.08	0.82	− 0.19	0.81	− 0.45	0.85	<.001	0.42
Teacher-reported aggression score	0.09	0.94	0.19	0.93	0.72	0.84	0.18	0.93	0.14	0.81	− 0.07	0.97	<.001	0.89
Teacher-reported hyperactivity score	− 0.09	0.95	0.06	0.95	0.46	0.85	− 0.15	0.94	− 0.04	0.87	− 0.11	1.02	<.001	0.89
Teacher-reported depression score	− 0.10	0.88	− 0.02	0.84	0.52	0.86	− 0.49	0.74	− 0.01	0.67	− 0.08	0.86	<.001	0.93
Teacher-reported anxiety score	− 0.15	0.90	− 0.10	0.88	0.34	0.83	− 0.76	0.64	0.04	0.77	− 0.03	0.82	<.001	0.67
Teacher-reported somatization score	− 0.16	0.86	− 0.12	0.92	0.34	0.91	− 0.64	0.57	− 0.21	0.72	− 0.06	0.84	<.001	0.19

*STB* suicidal thoughts and behavior, *SAU* school-as-usual, *SCP* standard coping power, *ICP* individual coping power, *MCP* mindfulness coping power, *IECP* internet-enhanced coping power, *Tier* 1 non-study universal intervention, *MLM p* values are based on multilevel models that account for study-level fixed effects and school-level random effects

### Procedure

Individual-level data from the original randomized trials were combined and analyzed as a single dataset and were harmonized using IDA (Hussong et al., 2020; Morgan-López et al., 2023). Parent-reported STBs were assessed across all 11 RCTs, with two to eight waves of data collection per study, representing approximately 1 to 7 years of follow-up. Teacher-reported STBs were assessed in 10 of 11 studies, with three to seven waves of data collection per study, again representing approximately 1 to 7 years of follow-up.

#### Intervention Conditions

In each of the 11 Coping Power (CP) trials, at least one intervention arm included the implementation of some form of CP. Ten of the studies included randomization to the original form of CP (i.e., Standard CP). Although all forms of CP address the same set of objectives, Standard CP (SCP) is longer and has more sessions (34 child group sessions, 16 parent group sessions) than the other CP variants. Two of the trials included randomization to Individual Coping Power (ICP), which has the same content as the **Standard** CP groups, but which is delivered one-on-one in contrast to being delivered in groups. Two trials included versions of CP which either (a) integrated online CP modules (i.e., Internet-Enhanced Coping Power [IECP]) or (b) mindfulness content (i.e., Mindfulness-Enhanced CP [MCP]). Two of the trials included randomization to an active non-CP universal intervention (i.e., Tier 1 intervention); nine of the trials included a school-as-usual comparison (SAU) condition. Each of the intervention conditions (i.e., Standard, Individual, Internet-Enhanced, and Mindfulness-Enhanced CP) and the active control condition were combined *across* trials, which created imbalance in covariates (see propensity score weighting below). Each of the CP conditions were compared to the SAU condition in our analyses. For additional details on the 11 RCTs, see Morgan-López et al. (2022).

### Measures

#### Covariates

Demographic variables (covariates) included grade in school, biological sex, race, and family income. Additional covariates included baseline measures of three internalizing (depression, anxiety, somatization) and three externalizing (conduct problems, aggression, hyperactivity) subdimensions. In addition to these listed covariates, additional predictors of differential item functioning included age at each timepoint and dummy variables representing each RCT, with the trial comparing Standard and Individual CP (Lochman et al., 2015) designated as the reference study. Measurement of internalizing and externalizing constructs is described below. Baseline descriptives are shown in Table 1.

#### Suicidal Thoughts and Behaviors

In nine of the RCTs, parents and teachers completed the Behavior Assessment System for Children (BASC/BASC-2; Reynolds & Kamphaus, 2004). In two of the RCTs, parents and teachers completed the Achenbach System of Empirically Based Assessment (ASEBA; Achenbach & Rescorla, 2004). Both assessment systems assess broadband mental health difficulties but have some key differences, including in construct conceptualization. For an overview of construct conceptualization and differences in the assessment system that may impact the current study, see an overview by McDaniel et al. (2023a). Across both assessment systems, five items (asked of both a parent and a teacher of the target child) were available that had STB content: 3 items from the BASC (Tries to hurt self; Says “I want to die” or “I wish I were dead”; Says, “I want to kill myself”) and 2 items from the ASEBA (Talks about killing themselves; Deliberately harms self or attempts suicide). These five items were evaluated for whether any of the items could be harmonized across assessment systems (i.e., similar item content to warrant a combined BASC/ASEBA item). We used expert rating procedures for semantic harmonization of transdiagnostic item content developed in McDaniel et al. (2023a). Of the 5-item pool, only the BASC “Says, ‘I want to kill myself’” item and the ASEBA “Talks about killing themselves” item were judged as harmonizable. The harmonized “Says, ‘I want to kill myself’/Talks about killing themselves” item had only 6.8% missingness. The remaining BASC items were treated as missing in studies with the ASEBA assessment and vice versa; this yielded a 4-item construct, but with an average item-level missingness of ~ 60% for non-harmonized items. This level of sparseness across assessment systems is not unusual for IDAs (Howe et al., 2024) and the STBs constructs meet the bare minimum of one item that overlaps across assessment systems (Bauer & Hussong, 2009). Missingness on the harmonized items was addressed using missing at random data approaches to scale score estimation (see e.g., Bauer & Hussong, 2009). Item responses for all items were recoded to binary (i.e., 0 = “Never/Not True”, 1 = all other responses) because of extremely sparse responding in the higher STB item categories. Parent- and teacher-reported STBs were modeled separately, as described in greater detail below. STB-related scale scores had already been estimated under zero-inflated mixture non-linear factor analysis (ZIM-NLFA; Saavedra et al., 2023; Wall et al., 2015) and were previously used in McDaniel et al. (2025).

#### Internalizing and Externalizing Problems

Internalizing and externalizing problems were assessed by the teacher-reported BASC and ASEBA. Following McDaniel et al. (2023a), we prioritized the construct conceptualization of the BASC system, as the BASC was collected across nine of 11 studies. We instructed expert raters to find the best exact or close conceptual match across the BASC and ASEBA, mapping the ASEBA items to the BASC scales. Raters matched conceptually paired scales, although raters could match ASEBA items outside the conceptually paired scales. Harmonization decisions proceeded in two phases: (a) if two raters agreed, the items were harmonized; (b) if the two raters did not agree, three additional raters considered the items. Items for which there was consensus were harmonized. Any ASEBA items not semantically harmonized to a BASC item were dropped from consideration in subsequent scale score estimation.

#### Conduct Problems

The Conduct scale on the BASC (*n*_*items*_ = 21) was conceptually matched with Rule Breaking Behavior (*n*_*items*_ = 12) on the ASEBA. The two raters agreed on 76.2% of the BASC item harmonization decisions; after the additional 3 raters resolved discrepancies, 9 items were deemed semantically harmonizable (e.g., “Breaks school rules” and “Breaks the rules”). The remaining 12 BASC items had missingness across studies collecting the ASEBA.

#### Aggression

The Aggression scale on the BASC systems (*n*_*items*_ = 20) was initially conceptually matched with Aggressive Behavior (*n*_*items*_ = 20) on the ASEBA system. Two raters agreed on 95.0% of the BASC item harmonization decisions. Following the 5-rater consensus phase, 11 items were deemed semantically harmonizable (e.g., “Defiant, talks back to staff” and “Talks back to teachers”). The remaining nine BASC items had missingness across studies collecting the ASEBA.

#### Hyperactivity

The Hyperactivity scale on the BASC (*n*_*items*_ = 18) did not have a parallel scale on the ASEBA system. However, items on the ASEBA Attention Problems (*n*_*items*_ = 26) scale were judged to have potential overlap with the BASC Hyperactivity scale. Raters were instructed to match ASEBA Attention Problems items with BASC Hyperactivity. Two raters agreed on 61.1% of the BASC item harmonization decisions. Following the 5-rater consensus phase, 8 items were deemed semantically harmonizable (e.g., “Acts without thinking” and “Impulsive or acts without thinking”). The remaining ten BASC items had missingness across studies collecting the ASEBA.

#### Depression (Excluding STB Items)

The Depression scale on the BASC (*n*_*items*_ = 13) was conceptually matched with the Withdrawn/Depressed scale (*n*_*items*_ = 8) on the ASEBA. Two raters agreed on 46.2% of the BASC item harmonization decisions. Following the 5-rater consensus phase, 6 items were deemed semantically harmonizable (e.g., “Complains of loneliness” and “Says ‘I have no friends’”). The remaining BASC items had missingness across studies collecting the ASEBA system.

#### Anxiety

The Anxiety scale on the BASC (*n*_*items*_ = 12) was conceptually matched with the Anxious/Depressed scale (*n*_*items*_ = 15) on the ASEBA. Two raters agreed on 91.7% of the BASC item harmonization decisions. Following the 5-rater consensus phase, five items were deemed semantically harmonizable (e.g., “Nervous, highstrung, or tense” and “Is nervous”). The remaining seven BASC items had missingness across studies administering the ASEBA.

#### Somatization

The Somatization scale on the BASC (*n*_*items*_ = 13) was conceptually matched with the Somatic Complaints scale (*n*_*items*_ = 9) on the ASEBA. Two raters agreed on 84.6% of the BASC item harmonization decisions. Following the 5-rater consensus phase, six items were deemed semantically harmonizable (e.g., “Headaches” and “Has headaches”). The remaining 7 BASC items had missingness across studies collecting the ASEBA.

### Analysis Plan

#### Tests of Dimensionality

A series of initial nonlinear confirmatory factor analysis models were fit in M*plus* Version 8 (Muthén & Muthén, 1998–2017) to assess unidimensionality of the harmonized measures. Models were estimated separately for teacher- and parent-reported scales. Models were estimated initially using mean-and-variance-adjusted weighted least squares (M*plus* WLSMV) for ordered categorical items to assess model fit. WLS approaches use summary information (i.e., category proportions, polychoric correlations) as input data against which fitted and saturated models can be compared and global fit statistics can be estimated (e.g., comparative fit index [CFI], root mean square error of approximation [RMSEA]).

#### STB Scale Score Estimation: Zero-Inflated Mixture NLFA

A series of zero-inflated mixture NLFA models (Saavedra et al., 2023; Wall et al., 2015) were used to assess differential item functioning (DIF) on item intercepts^1^; intercept DIF is present if an item differs in STB item endorsement rates across a covariate, controlling for the same level of underlying latent STB. Intercept DIF was explored across grade in school, biological sex, race, and family income, study and study wave for each measure. Following Gottfredson et al. (2019), scale scores were estimated separately for each reporter, with the scale of the factor set to *Normal* (0, 1) at baseline. Greater detail on methodology for latent variable scale scoring in the presence of zero-inflation is described in McDaniel et al. (2025) and Saavedra et al. (2023).

#### Internalizing and Externalizing Scale Score Estimation: Moderated NLFA

Excluding STB items, we fitted a series of MNLFA models (Bauer, 2017, 2023) for each individual internalizing and externalizing subdimension, separately for parent and teacher reports. We examined intercept and factor loading DIF in separate models for each item across the set of predictors of DIF described above as well as dummy variables reflecting study membership. DIF parameters that were significant at *p* ≤.001 (Bonferroni adjusted α level to control for multiple comparisons) were retained for a global MNLFA model. For those items for which tests of DIF were significant in the global model, specific DIF parameters were retained for the final MNLFA scale score estimation model. Scale scores estimated under MNLFA for each internalizing and externalizing subdimension were then output using the M*plus* SAVEDATA command for later analysis.

#### Propensity Score Weighting

Propensity score weighting was employed to address unbalanced assignment to intervention conditions that resulted from combining data *across* RCTs. Intervention conditions were randomized within each original trial, but differences in study populations and/or inclusion criteria across trials could compromise covariate balance in the IDA (Brincks et al., 2018; Hien et al., 2023; Morgan-López et al., 2022; Saavedra et al., 2021). The covariates listed above, in addition to (a) baseline scores for STBs, (b) baseline scores for each mediator, (c) dummy variables for each trial, and (d) random effects for each school were examined for their potential relations to both treatment assignment and baseline levels of both the mediators and outcomes for potential inclusion in PSW models (Cook et al., 2020; Steiner et al., 2010). Only covariates that both (a) differ between the treatment and control groups at baseline plus the mediator and (b) are related to the outcome variable can potentially serve as confounders and bias the estimate of the mediational effect. The only covariates that met these criteria were baseline scores for each mediator, study dummy variables, and school-level random effects; other covariates (e.g., biological sex, race) were either balanced across conditions or did not jointly predict treatment assignment and baseline mediators/outcomes. We used multilevel multinomial logistic regression modeling in SAS Proc GLIMMIX to estimate inverse probability of treatment weights to balance covariates across the five intervention conditions (Standard CP, Individual CP, Internet-Enhanced CP, Mindfulness-Enhanced CP, active control condition) versus the SAU control condition.

#### Outcome Models

Outcome models were fitted using M*plus* version 8 (Muthén & Muthén, 1998–2017). We specified a series of propensity score-weighted parallel process mediation models (Cheong et al., 2003; Di Maria & Didelez, 2024) across the two outcomes (teacher- and parent-reported STBs) and six intermediate endpoints treated as mediators (aggression, conduct problems, hyperactivity, depression, anxiety, somatization). Given that the data came from 11 separate randomized trials, clustering of the data is an issue. Following Kreft and de Leeuw (1998) and McNeish and Kelley (2019), we used study-level dummy variables as predictors of baseline levels of mediators and outcomes to address clustering. The small number of study units (*n* = 11) precluded the use of random effects in multilevel modeling which requires at least 30 study units (McNeish & Kelley, 2019). We implemented this analysis using cluster-robust standard error adjustments under M*plus* “TYPE = COMPLEX.” For the two active intervention conditions where this was possible (i.e., Standard CP, Individual CP), these conditions were disaggregated into school-level proportions (e.g., the proportion of participants in each school receiving Standard CP) and (residual) individual-level variability within-schools for both of these conditions. We used this form of observed (as opposed to latent) multilevel covariate disaggregation (Lüdtke et al., 2008) because intervention assignment is known. The Mindfulness CP (MCP) condition was measured only at the individual-level, as there was no variation in the mean proportion of youth randomized to MCP versus school-as-usual (SAU) across the five schools in Boxmeyer et al. (2021). The Internet-Enhanced CP (IECP) condition was aggregated up to the school-level (with no individual-level variability) for the four participating schools, given the school-randomized design in this trial (Lochman et al., 2017). In addition to overall models estimated with all participants, separate stratified models were fit for (a) boys and girls and (b) elementary school trials and middle/high school trials.

#### Estimation of Mediation Effects

Pre-registered plans for estimation of mediation effects for this study (Morgan-López et al., 2022) included both traditional and potential outcomes estimates for mediated effects (MacKinnon, in press; MacKinnon & Pirlott, 2015; VanderWeele & Vansteelandt, 2009). According to MacKinnon et al. (2020), traditional mediation effects can be considered causal when the following assumptions are met: (1) no spillover effects of treatment, (2) no exposure-mediator interaction effects on the outcome, and (3) sequential ignorability for the effect of the exposure on the mediator and for the effect of the mediator on the outcome (see, e.g., Saavedra et al., 2023). Tests of treatment-mediator interaction effects were nonsignificant across all models and intervention conditions (all *p*s >.12). In situations where treatment-mediator interactions are nonsignificant, traditional mediation effects and potential outcomes mediation effects are equivalent (MacKinnon et al., 2020). Following MacKinnon (in press) and Shrout and Bolger (2002), we used bias-corrected percentile bootstrapping procedures to estimate the empirical confidence intervals for the mediated effects (BCB; Efron & Tibshirani, 1994; Shrout & Bolger, 2002), as implemented under the M*plus* “CINTERVAL(BCBOOT)” command with 1000 bootstrap resamples per model. Three considerations informed our use of BCB, each of which could compromise statistical inferences in this study with parametric inference: (1) non-normality in the sampling distribution of the mediated effect (αβ), even when the sampling distributions of the individual parameters (α and β) are normal (MacKinnon et al., 2002); (2) non-normality in STB outcome distributions (i.e., latent variable “zero-inflation”; Saavedra et al., 2023), which can distort that sampling distributions of individual regression coefficients (i.e., α and β themselves; Kelley, 2005); and (3) small cell sizes for MCP and ICP (*n*s ~ 50 each), which can impact the precision of estimates for α and β. We elaborate on the use of bootstrapping and traditional procedures in the “Discussion” section.

## Results

### STB Scale Score Estimation: Zero-Inflated Mixture NLFA

McDaniel et al. (2025) using ordinal item CFAs found evidence for the unidimensionality for of harmonized parent- (CFI =.99, RMSEA =.041) and teacher-reported (CFI =.99, RMSEA =.035) STB measures.

#### Internalizing and Externalizing Scale Score Estimation: Moderated NLFA

Preprocessing of data for the unidimensional factor models include collapsing sparse cells across categories and estimating polychoric correlations to identify problematic items (e.g., collinearity). The remaining items were included in an initial unidimensional model run separately for subsamples of studies collecting data using a particular assessment system (i.e., studies collecting BASC-1, BASC-2, ASEBA). Modification indices were utilized to identify issues of local dependence. Problematic items were pruned, prioritizing retention of items that were semantically harmonized. Fit statistics for final models for each construct are reported in Tables 2 and 3 specific to the ASEBA/BASC 1/BASC 2 assessment systems. Final item parameters across BASC/ASEBA harmonized measures from which MNLFA scale scores were estimated for each subdimension are included in Supplemental Tables 1–6; in addition to DIF identified by biological sex and age, DIF by study fixed effects was identified that was likely as a function of DIF by measure (i.e., studies that used the ASEBA; Lochman & Wells, 2002) or DIF by developmental stage for the CP trials implemented in middle and high schools (Bradshaw et al., 2025; Thomas et al., 2021).

**Table 2:** Fit statistics for internalizing subdimensions by assessment system

Subdimension	Depression			Anxiety			Somatization		
	ASEBA	BASC-1	BASC-2	ASEBA	BASC-1	BASC-2	ASEBA	BASC-1	BASC-2
Sample size	424	1523	1184	171	1523	1183	171	1523	1184
Number of items	6	7	7	5	7	6	4	8	8
CFI	1	.99	.99	1	.99	.99	.99	.98	.99
RMSEA (90% CI)	.000 (.000.052)	.071 (.060.083)	.049 (.035.063)	.000 (.000.088)	.067 (.056.079)	.050 (.033.067)	.000 (.000 0.167)	.074 (.065.084)	.028 (.013.041)

*ASEBA* Achenbach System for Empirically Based Assessment, *BASC* behavioral assessment system for children, *CFI* comparative fit index, *RMSEA* root mean square error of approximation, *CI* confidence interval

**Table 3:** Fit statistics for externalizing subdimensions by assessment system

Subdimension	Conduct problems			Aggression			Hyperactivity		
	ASEBA	BASC-1	BASC-2	ASEBA	BASC-1	BASC-2	ASEBA	BASC-1	BASC-2
Sample size	343	1282	945	343	1282	945	126	1282	945
Number of items	6	10	9	7	5	6	5	9	5
CFI	.99	.98	.99	.99	.99	.99	.98	.98	.99
RMSEA (90% CI)	.064 (.029,.099)	.042 (.033,.050)	.053 (.042,.064)	.095 (.070,.121)	.042 (.020,.065)	.042 (.022,.063)	.000 (.000,.174)	.078 (.069,.087)	.073 (.048,.098)

*ASEBA* Achenbach System for Empirically Based Assessment, *BASC* behavioral assessment system for children, *CFI* comparative fit index, *RMSEA* root mean square error of approximation, *CI* confidence interval

#### Propensity Score Weights and Balance Checks

Following weighting, standardized mean differences at baseline did not exceed *d* =|.10| and were not statistically significant, mimicking the covariate balance of a multi-arm, multi-site randomized experiment. Significant covariates that were included in the final parent-reported STB models included baseline levels of parent-reported STBs and race/ethnicity; the corresponding teacher-reported STB models included baseline levels of teacher-reported STBs and race/ethnicity (McDaniel et al., 2025). Unweighted descriptive statistics are shown in Table 1.

#### Model Outcomes^2^

Effects of mediator slopes on STB slopes (β), effects of intervention conditions on mediator slopes (α), mediated effects of the effects of CP on STBs (αβ), and direct effects (τ’) are reported below for the full sample and then for biological sex and grade level subgroups. We conducted a separate analysis for each mediator which provides an estimate of its effect. We did not pursue multiple mediator models; the unique effect of each construct over and above the set of other constructs is considerably more difficult to interpret and could not easily be utilized for the design of future interventions.

#### CP Effects on STBs through Aggression: Full Sample

Reductions over time in aggression were associated with reductions in teacher-reported STBs ($\widehat{\beta }$=.093 (.025), *t* = 3.76, *p* <.001, 2-tailed 95% CI [.056,.154]), but not with changes in parent-reported STBs (*p* =.97). The MCP (${\widehat{\alpha }}_{MCP}$= −.207 (.109), *t* = − 1.89, *p* =.06*, 2-tailed 95% CI [−.532, −.026]), school-aggregated SCP (${\widehat{\alpha }}_{SCP}$= −.069 (.040), *t* = − 1.72, *p* =.08**, 2-tailed 95% CI [−.165,.002]), and school-aggregated ICP intervention conditions (${\widehat{\alpha }}_{ICP}$= −.134 (.045), *t* = − 2.98, *p* =.003, 2-tailed 95% CI [−.239, −.055]); all CP interventions showed steeper reductions in aggression compared to the school-as-usual control condition. Tests for mediation of CP’s effects (see Table 4) on teacher-reported STBs were all significant for MCP (${\widehat{\alpha \beta }}_{MCP}$= −.019 [−.077, −.005]), school-aggregated SCP (${\widehat{\alpha \beta }}_{SCP}$= −.006 [−.019, −.001]), and school-aggregated ICP (${\widehat{\alpha \beta }}_{ICP}$= −.012 [−.028, −.006]). Direct effects favoring *individual*-level ICP (${\widehat{\tau {\prime}}}_{ICP}$= −.048 (.016), *t* = − 2.93, *p* =.003, 2-tailed 95% CI [−.080, −.014]) suggest additional benefits on teacher-reported STBs of ICP that may operate through other unmeasured mediators. A direct effect favoring IECP (${\widehat{\tau {\prime}}}_{IECP}$= −.102 (.045), *t* = − 2.28, *p* =.02, 2-tailed 95% CI [−.175,.009]) suggested that IECP does not impact teacher-reported STBs through aggression (nor any other measured intermediate endpoint; see below). Also for IECP, the discrepancy in results between the significant *parametric t* value at *p* =.05 versus 0 being in the 95% bias-corrected bootstrap confidence interval suggests the bias correction for small cells (*n* ~ 50) has an impact on inferences regarding statistical significance for IECP. Note that the overall effect sizes for IECP effects on STBs were strong (*d* = −.50; McDaniel et al., 2025). Direct effects in an *unpredicted* direction for MCP (${\widehat{\tau {\prime}}}_{MCP}$=.134 (.040), *t* = 3.32, *p* =.001, 2-tailed 95% CI [.078,.284]), taken in the context of the favorable mediation effect above, suggest that STB trajectories can be worse for youth exposed to MCP *whose aggressive behavior did not improve.* This finding is in line with inconsistent mediation effects in which the mediated and direct effects are opposite in sign (MacKinnon, 2000; MacKinnon et al., 2000; Saavedra et al., 2023).

**Table 4:** Overall mediation model estimates and 95% confidence intervals

	Standard CP			Individual CP			Mindful CP			Internet-enhanced CP		
Mediator/Outcome	ab	LCL	UCL	ab	LCL	UCL	ab	LCL	UCL	ab	LCL	UCL
Aggression/PRS	− 0.001	− 0.009	0.001	− 0.001	− 0.011	0.003	− 0.002	− 0.019	0.005	0.000	− 0.010	0.008
Aggression/TRS	− ***0.006***	− ***0.019***	− ***0.001***	− ***0.012***	− ***0.028***	− ***0.006***	− ***0.019***	− ***0.077***	− ***0.005***	− 0.001	− 0.016	0.061
Conduct Problems/PRS	0.001	− 0.002	0.005	0.002	− 0.005	0.010	0.003	− 0.008	0.014	0.000	− 0.007	0.007
Conduct Problems/TRS	− ***0.006***	− ***0.015***	− ***0.004***	− ***0.013***	− ***0.033***	− ***0.004***	− ***0.018***	− ***0.044***	− ***0.005***	0.000	− 0.012	0.033
Hyperactivity/PRS	− 0.001	− 0.006	0.003	− 0.001	− 0.010	0.005	− 0.003	− 0.019	0.013	− 0.001	− 0.031	0.011
Hyperactivity/TRS	− 0.003	− 0.009	0.000	− ***0.006***	− ***0.018***	− ***0.001***	− ***0.015***	− ***0.038***	− ***0.003***	− 0.006	− 0.034	0.027
Depression/PRS	0.003	− 0.001	0.016	0.005	− 0.003	0.024	0.010		0.056	0.003	− 0.004	0.042
Depression/TRS	0.002	− 0.003	0.014	0.002	− 0.007	0.018	0.004	− 0.010	0.049	0.001	− 0.007	0.028
Anxiety/PRS	0.002	− 0.001	0.010	0.004	− 0.001	0.015	0.008	− 0.003	0.035	− 0.003	− 0.057	0.004
Anxiety/TRS	− 0.001	− 0.006	0.001	− 0.002	− 0.014	0.001	− 0.005	− 0.023	0.003	0.001	− 0.002	0.041
Somatization/PRS	0.000	− 0.002	0.003	0.000	− 0.003	0.008	0.001	− 0.013	>0.024	0.000	− 0.036	0.010
Somatization/TRS	0.000	− 0.003	0.001	0.000	− 0.006	0.001	− 0.002	− 0.013	0.008	0.001	− 0.005	0.021

*PRS* parent-reported suicidality, *TRS* teacher-reported suicidality

#### CP Effects on STBs through Aggression: Subgroup Analyses

Grade-level stratified sub-analyses suggested that the mediated effects through reductions in aggression for MCP (${\widehat{\alpha \beta }}_{MCP}$= −.018 [−.061, −.001]) and school-aggregated ICP (${\widehat{\alpha \beta }}_{ICP}$= −.012 [−.028, −.004]) were driven by the elementary school trials. Biological sex-stratified sub-analyses suggested that boys drove the mediated effects through reductions in aggression for MCP (${\widehat{\alpha \beta }}_{MCP}$= −.022 [−.064, −.003]), school-aggregated SCP (${\widehat{\alpha \beta }}_{SCP}$= −.010 [−.030, −.002]), and school-aggregated ICP (${\widehat{\alpha \beta }}_{ICP}$= −.018 [−.044, −.007]); the corresponding effects for girls were all nonsignificant (all *p*s >.17). Formal tests of conditional mediation (Fairchild & MacKinnon, 2009) showed no differences between boys and girls nor across grade level for this and all other formal tests for conditional mediation across subgroups.

#### CP Effects on STBs Through Conduct Problems: Full Sample

Reductions over time in conduct problems were associated with reductions over time in teacher-reported STBs ($\widehat{\beta }$=.086 (.022), *t* = 3.97, *p* <.001, 2-tailed 95% CI [.054,.136]), but not parent-reported STBs (*p* =.61). The MCP (${\widehat{\alpha }}_{MCP}$= −.212 (.095), *t* = − 2.24, *p* =.02**, 2-tailed 95% CI [−.347,.008]), school-aggregated SCP (${\widehat{\alpha }}_{SCP}$= −.068 (.036), *t* = − 1.91, *p* =.06**, 2-tailed 95% CI [−.144,.002]), and school-aggregated ICP interventions (${\widehat{\alpha }}_{ICP}$= −.146 (.050), *t* = − 2.92, *p* =.003, 2-tailed 95% CI [−.242, −.043]) all showed steeper reductions in conduct problems compared to the school-as-usual control. Tests for mediation of CP’s effects on teacher-reported STBs were significant for MCP (${\widehat{\alpha \beta }}_{MCP}$= −.018 [−.044, −.005]), school-aggregated SCP (${\widehat{\alpha \beta }}_{SCP}$= −.006 [−.015, −.001]), and school-aggregated ICP (${\widehat{\alpha \beta }}_{ICP}$= −.013 [−.033, −.004]). As with aggressive behavior above (and across all other models below), direct effects favoring *individual*-level ICP and IECP and direct effects in an *unpredicted* direction for MCP remained.

#### CP Effects on STBs Through Conduct Problems: Subgroup Analyses

Grade-level stratified sub-analyses suggested that the mediated effects through reductions in aggression for MCP (${\widehat{\alpha \beta }}_{MCP}$= −.016 [−.039, −.003]) and school-aggregated ICP (${\widehat{\alpha \beta }}_{ICP}$= −.011 [−.033, −.003]) were driven by the elementary school trials. Biological sex-stratified sub-analyses suggested that boys drove the mediation effects through reductions in conduct problems for school-aggregated SCP (${\widehat{\alpha\beta}}_{SCP}$= −.009 [−.028, −.002]), and school-aggregated ICP (${\widehat{\alpha \beta }}_{ICP}$= −.019 [−.053, −.008]), whereas girls drove the corresponding mediation effect favoring MCP (${\widehat{\alpha \beta }}_{MCP}$= −.024 [−.054, −.006]).

#### CP Effects on STBs Through Hyperactivity: Full Sample

Reductions over time in hyperactivity were associated with reductions in teacher-reported STBs ($\widehat{\beta }$=.086 (.022), *t* = 3.97, *p* <.001, 2-tailed 95% CI [.054,.136]), but not parent-reported hyperactivity (*p* =.67). The MCP (${\widehat{\alpha }}_{MCP}$= −.256 (.078), *t* = − 3.28, *p* =.001, 2-tailed 95% CI [−.393, −.096]) and school-aggregated ICP interventions (${\widehat{\alpha }}_{ICP}$= −.112 (.039), *t* = − 2.85, *p* =.004, 2-tailed 95% CI [−.192, −.037]) showed steeper reductions in hyperactivity compared to the school-as-usual control. Tests for mediation of CP’s effects on teacher-reported STBs were significant for MCP (${\widehat{\alpha \beta }}_{MCP}$= −.015 [−.038, −.003]) and school-aggregated ICP (${\widehat{\alpha \beta }}_{ICP}$= −.013 [−.033, −.004]).

#### CP Effects on STBs Through Hyperactivity: Subgroup Analyses

Grade-level stratified sub-group analyses suggested that the mediation effects through reductions in hyperactivity for MCP (${\widehat{\alpha \beta }}_{MCP}$= −.016 [−.046, −.002]) and school aggregated ICP (${\widehat{\alpha \beta }}_{ICP}$= −.006 [−.021, −.001]) interventions were driven primarily by the elementary school trials, whereas a corresponding mediation effect for the middle/high school trials (but not for the overall sample, nor the elementary school trials subgroup) was found for SCP treatment (${\widehat{\alpha\beta}}_{SCP}$= −.015 [−.058, −.004]). Despite overall mediation effects that were significant, the corresponding mediation effects for the boys and girls subgroups were non-significant.

#### CP Effects on STBs Through Depression: Full Sample

The MCP (${\widehat{\alpha }}_{MCP}$= −.229 (.106), *t* = − 2.16, *p* =.03, 2-tailed 95% CI [−.495, −.056]), school-aggregated SCP (${\widehat{\alpha }}_{SCP}$= −.079 (.032), *t* = − 2.45, *p* =.014, 2-tailed 95% CI [−.144, −.015]), and school-aggregated ICP interventions (${\widehat{\alpha }}_{ICP}$= −.128 (.052), *t* = − 2.46, *p* =.014, 2-tailed 95% CI [−.233, −.021]) showed steeper reductions in depression compared to the school-as-usual control condition; however, changes in depression were not associated with changes in teacher-reported STBs (*p* =.65) nor parent-report STBs (*p* =.35), suggesting that depression did not mediate effects of any CP intervention on STBs.

#### CP Effects on STBs Through Depression: Subgroup Analyses

Despite the lack of overall mediation, a mediation effect was observed for school-aggregated SCP in the middle/high school trials (but not in the elementary school trials) (${\widehat{\alpha \beta }}_{SCP}$= −.016 [−.043, −.003]). No mediation effects were observed in the boys or girls subgroups.

#### CP Effects on STBs Through Anxiety: Full Sample

The MCP (${\widehat{\alpha }}_{MCP}$= −.242 (.103), *t* = − 2.36, *p* =.02, 2-tailed 95% CI [−.446, −.015]) and school-aggregated ICP interventions (${\widehat{\alpha }}_{ICP}$= −.107 (.054), *t* = − 1.98, *p* =.04, 2-tailed 95% CI [−.224, −.003]) showed steeper reductions in anxiety compared to the school-as-usual control condition; however, as with depression, neither reductions in teacher-reported STBs (*p* =.38) nor parent-reported STBs (*p* =.25) were linked to reductions over time in anxiety. There was no evidence of mediation of the effects of any CP intervention on STBs through anxiety *in the overall sample*.

#### CP Effects on STBs Through Anxiety: Subgroup Analyses

Despite the lack of overall mediation, mediation effects through reductions in anxiety were observed for (a) school-aggregated SCP in the middle/high school trials (but not in the elementary school trials) (${\widehat{\alpha \beta }}_{SCP}$= −.010 [−.036, −.002]) and (b) Mindful CP for girls (but not boys; ${\widehat{\alpha \beta }}_{MCP}$= −.024 [−.068, −.006])

#### CP Effects on STBs Through Somatization

There were no differences between the CP intervention conditions and school-as-usual control condition on somatization trajectories, nor were changes in somatization associated with changes in teacher- (*p* =.71) nor parent- (*p* =.91) reported STBs, consistent with a lack of mediation of any CP intervention on STBs through somatization.

## Discussion

Much of the early work in distal STB prevention focused on secondary analyses of single data sets of early interventions that did not target STBs per se on downstream reductions in suicide-specific risk markers (Bai et al., 2025; Connell et al., 2016; Vidot et al., 2016; Sandler et al., 2016; Wilcox et al., 2008). More recently, there has been an emphasis on IDA studies of distal crossover intervention effects on STBs (Pearson & Sims, 2023; Reider & Sims, 2016). IDAs have the advantage (among others; see Curran et al., 2008) of increasing statistical power for low base rate outcomes such as STBs and permitting indirect comparisons between intervention conditions that were never part of the same trial (Morgan-López et al., 2022). Results from initial IDA studies have been promising (Connell et al., 2023; McDaniel et al., 2025; Musci et al., 2023), but there are still gaps in this relatively nascent literature. These gaps include (a) limited emphasis on STB crossover effects from *externalizing*-focused interventions compared to more heavily researched crossover effects of internalizing-focused interventions, (b) a dearth of IDA studies examining mediation effects on STBs via the original targets of the intervention, (c) a lack of specificity regarding the mechanisms of action operating through internalizing and externalizing subdimensions, and (d) a lack of consideration of potential biological sex- and developmental-specificity in these pathways. The current study helps fill these gaps and extends the work of McDaniel et al. (2025) on an 11-study IDA of Coping Power to assess indirect effects of different forms of CP on teacher- and parent-reported STBs.

### Findings by CP Intervention

#### Group/Standard CP Effect Summary

Mediated effects favoring SCP were observed on STBs largely through reductions in aggression, conduct problems, and depression, with boys in particular benefitting more through aggression and conduct problems. Older youth benefited through reductions in hyperactivity, depression, and anxiety. These effects were generally school-level effects, suggesting that STB risk decreases when SCP is delivered to a higher proportion of children within the school (i.e., school-randomized SCP versus individual SCP).

#### Individual CP

Mediated effects of ICP on STBs were observed largely through reductions in aggression, conduct problems, hyperactivity, depression, and anxiety. Boys in elementary school benefitted by reductions in aggression and conduct problems, whereas broader elementary school effects operated through reductions in hyperactivity. Direct effects of ICP on STB remained, suggesting that ICP also operates through other as yet-to-be-modeled mediators such as CP-specific mechanisms of action (e.g., emotion regulation, hostile attribution bias).

#### Mindful CP

Mediated effects favoring MCP were observed on STBs through reductions in aggression, conduct problems, hyperactivity, depression, and anxiety. Boys in elementary school in particular benefitted more through reductions in aggression and conduct problems, whereas girls benefitted through reductions in conduct problems and anxiety. However, direct effects suggest inconsistent mediation effects (MacKinnon et al., 2000), in which STBs can *increase* for youth in cases where MCP does not improve on intermediate endpoints/mediators.

#### Internet-Enhanced CP

For IECP, there were no significant mediation effects at all. Favorable direct effects on reductions in STB were significant using a conventional *p* value, as consistent with total effect findings favoring IECP (McDaniel et al., 2025). However, zero was within the bias-corrected bootstrap confidence intervals, and this discrepancy between *p* values and BCB CIs may exist for at least two reasons: (a) non-normality in the distribution the STB outcome, (b) adjustments for bias-correction due to small sample size, or both (Kelley, 2005).

#### Findings by Intermediate Endpoint/Mediator

##### Externalizing as a Mediator

Reductions in hyperactivity led to reduced risk for STB, though only for MCP and ICP. It appears that when there are intervention-produced reductions in hyperactivity, and in its associated features such as impulsivity and lack of planning for future consequences, children are better able to inhibit suicidal thoughts and behaviors. Subgroup analyses indicated that the links between aggression and conduct problems with STB were strongest for boys and when the intervention was provided in the elementary school years. Stronger associations have been found between externalizing behavior and suicide attempts for boys than for girls (Brent et al., 1999; Joseph et al., 2024; Rhodes et al., 2014). The current study’s finding that reductions in externalizing behavior have more influence on STB for boys is thus consistent with prior research finding stronger relationships between externalizing behavior and suicidal outcomes for boys.

Boys with externalizing problems are most at risk for this outcome; in theory, boys stand to benefit most from reductions in the risk factors involving externalizing behavior (Morgan-López et al., 2022), potentially due to intervention-activated changes in how peers and parents respond to them. Peer relations are dynamic; boys’ aggressive behavior can elicit antagonistic behavior from peers which can then have powerful eliciting effects on the boys subsequent functioning. In a related way, Zheng and colleagues (2025) have found that children’s aggressive behavior can lead to them being more physically victimized, which in turn indirectly leads to more non-suicidal self-injury behaviors, but they found this relationship only existed for boys. This pattern is consistent with findings that boys who are bully-victims (are both aggressive and are victimized by others) have high rates of suicide attempts (Kerr et al., 2017). The Coping Power intervention, delivered in groups or individually, is designed to reduce children’s aggressive behavior by improving their emotion regulation and social problem-solving strategies, and to enhance their social skills and focus on positive peer relationships, and these changes could reduce the propensity for the boys to be around children who victimize their peers and to have enhanced coping skills when they do encounter efforts to bully them.

Decades of research have found that harsh parenting practices can serve to start and maintain children’s aggressive behavior, and these interconnected risk factors could then play a role in affecting boys’ STB. Children who received punitive parenting practices when they were in latter years of elementary school have been found to produce increases in children’s subsequent conduct disorder and social aggression, which in turn then predicted their suicidal ideation and attempts in early adolescence, but this mediation path was only evident for boys (Kingsbury et al., 2020). Harsh parenting has been found to have stronger reactive effects on aggressive behavior for boys, than for girls (Kerr et al., 2004), and intervention-produced reductions in negative parenting practices could have the longitudinal effect of reducing boys’ aggression and conduct problems and then their risk for STB. Coping Power’s parent component, delivered alongside standard Coping Power for the youth, is designed to promote positive parenting and reduce harsh and inconsistent parenting, and thus could be expected to affect this mediation path. Although Individual Coping Power was not delivered with the parent component, we have found that children’s reductions in conduct problems in ICP led to reductions in caretaker depression (Saavedra et al., 2025), which, speculatively, could in turn lead to reduced negative parenting behaviors as has been observed in prior research (e.g., Barry et al., 2009) and ultimately to STB effects.

That the linkage between reductions in externalizing behavior and subsequent STB is stronger when the intervention was delivered in the elementary rather than in the middle school years is consistent with findings that psychosocial interventions can more readily reduce externalizing problems during the preadolescent years than during adolescence (Conduct Problems Prevention Research Group, 2020). The internal and external mechanisms affecting child externalizing behaviors are expected to be more malleable and open to change during preadolescence than during adolescence, when other powerful risk factors (e.g., deviant peer group influences; Dishion et al., 1999) begin to predominate.

#### Internalizing as Mediator

Although CP interventions led to reductions in internalizing problems, the role of the three internalizing subdimensions as mediators of STB outcomes was less consistent compared to the externalizing subdimensions. Of the internalizing subdimensions, depression emerged as the strongest mediator, with several CP variants, particularly SCP and ICP, reducing teacher-reported STBs through decreases in (non-STB) depression. These effects were more pronounced in middle and high school students, suggesting that depression-related risk for suicidality becomes more salient in later developmental stages. In contrast, several CP intervention variants had no significant *overall* mediation effects through anxiety on STBs.

Biological sex-specific analyses further highlighted differences in internalizing pathways. MCP was effective for girls, with reductions in anxiety mediating lower STB risk, a pattern not observed overall nor specifically in boys. These findings are in line with literature that suggests girls are more likely to experience STBs via internalizing stress (Dunning et al., 2019) with reductions in anxiety mediating lower STB risk. The finding that MCP reduced STB risk for girls through decreases in anxiety likely reflects both the specific mechanisms targeted by mindfulness and the way anxiety is observed and reported by teachers. Conceptually, mindfulness interventions directly address key components of anxiety—emotional regulation, attentional control, and stress tolerance—skills that can be particularly relevant for girls. MCP uniquely teaches strategies that indirectly manage anxious thoughts and physiological arousal, facilitating disruption of the anxiety-suicidality pathway. Given that these effects were detected through teacher-reported anxiety and STBs, MCP appears to lead to observable external changes in classroom behavior, such as reduced avoidance, increased engagement, or fewer distress-related disruptions. Teachers may be less likely to perceive more internal aspects of anxiety, such as excessive worry or rumination. If child self-reports had been used, the relationship might have been even stronger (and perhaps also observed among boys), as youth are the best informants of their own anxious thoughts. Our findings highlight both the unique benefits of mindfulness in addressing anxiety-related suicide risk for girls and the importance of considering the sensitivity with which different informants perceive changes in behavior and internal thoughts. The absence of significant mediation effects for somatization further underscores the need to differentiate among internalizing subdimensions; broad internalizing constructs may obscure specific risk and protective factors in suicide prevention (Saavedra et al., 2025), although self-reported somatization may have also yielded different results.

### A Methodological Caution: Using Bootstrapping Methods with Stbs

A particular caution from this study involves our recommendation that investigators examining STBs (Connell et al., 2023; Musci et al., 2023; Seidman et al., 2025) and other low base rate latent variables more broadly (Saavedra et al., 2023; Wall et al., 2015) utilize bootstrapping or other resampling approaches. Bootstrapping approaches have long been advocated in mediation analysis (e.g., MacKinnon et al., 2004, 2007; Shrout & Bolger, 2002) because the sampling distribution of mediated effects is typically non-normal. Traditional parametric approaches (e.g., Sobel, 1982) depend on an asymptotic derivation that assumes normality and do not perform well at sample sizes characteristic of prevention research. Although there are parametric methods for null hypothesis testing that explicitly account for skew and kurtosis in the distribution of the mediated effect (MacKinnon et al., 2004; Meeker et al., 1981), bootstrapping (a) estimates the sampling distribution empirically without the need for prior theoretical knowledge regarding the shape of the sampling distribution of the parameter of interest (Efron & Tibshirani, 1994), (b) can accommodate non-symmetric sampling distributions, and (c) is now more readily and easily accessible in commercial (e.g., SAS, M*plus*, Stata) and open source software (e.g., “boot” and “bootstrap” packages in R).

In mediation analysis more generally, concerns regarding non-normality in the distribution of mediation effects emerge even in models in which the (a) the intervention effect on the mediator ($\widehat{\alpha })$ and (b) the mediator’s effect on the outcome $(\widehat{\beta })$ each are normally distributed. However, latent STBs had the majority of observations concentrated at the low end of the distribution. In our study, distributional issues likely led to a number of effects where, for individual $\widehat{\alpha }$ and $\widehat{\beta }$ paths, different inferences were reached under parametric inference (i.e., conventional *p* values) versus non-parametric inferences under bias-corrected bootstrapped CIs. Small sample size was likely an issue; the estimate of the bias correction factor in BCB can also have some impact on statistical inference (Kelley, 2005). We encourage all investigators within the STB space to consider making regular use of bootstrapping and other resampling methodologies given that the latent and continuous measures of STBs are by their nature extremely skewed and kurtotic.

### Limitations

While the present study has several notable strengths, key limitations should be acknowledged. One limitation of the current study is the reliance on latent growth mediation (LGM) models (Cheong et al., 2003; Di Maria & Didelez, 2024), which use linear trajectories of mediators and outcomes over time but do not account for dynamic mediation processes that can be modeled in data sets with many measurement waves. This issue is particularly relevant for two reasons. First, prior dynamic mediation research on CP interventions suggests that most of the effects—at least for non-STB outcomes—occur within the first two years post-intervention (see Saavedra et al., 2025 which had 8 measurement waves). In the present integrated CP dataset, many of the studies had a limited number of measurement waves which precluded the use of these approaches that can more closely study the relation between intermediate endpoints and STBs during the time interval in the process where change would be expected to occur. LGM mediation can be applied to any study with at least three time points, giving it the advantage that it can be applied to each of the 11 studies in the IDA, permitting estimation of average mediational effects for mediators and STBs for all participants. However, the conclusions are based on the trajectories of the variables which might not be optimal. Alternative dynamic approaches study whether elevations in externalizing and internalizing precede suicidal thoughts, behaviors, and attempts values, clearly establishing temporal precedence. However, temporal precedence between mediators and STBs in this current study depends on the *conceptual* links between elevations in externalizing and internalizing that have been shown to precede suicidal thoughts, behaviors and attempts (Brent et al., 1999; Joseph et al., 2024; Kahn et al., 2024; Rhodes et al., 2014; Schaefer et al., 2018).

Related are concerns regarding sequential ignorability for (a) effects of CP on the mediators, (b) effects of CP on STB outcomes, and (c) links between. We addressed intervention-related confounding, given this study likely *created* confounding by combining intervention conditions across RCTs, via propensity score weighting; however, PSW cannot balance unobserved confounding in ways that randomization does. Sequential ignorability linking internalizing/externalizing to STBs outcomes is much more difficult, given most studies do not randomize participants to different levels of mediators (but see West & Aiken, 1997 for exceptions). While we examined whether the internalizing/externalizing-to-STBs effects differed by intervention conditions, consistent with examining potential outcomes for mediation (MacKinnon et al., 2020), other confounders were not examined here. Other factors that could strengthen causal inferences for links between internalizing/externalizing and STBs include (a) modeling covariation between changes in internalizing/externalizing subdimensions (i.e., multiple mediator growth models), (b) incorporation of CP mechanisms of action into the causal chain (e.g., hostile attribution bias, emotion regulation; Lochman et al., 2015), (c) and direct modeling of specific post-intervention confounders (e.g., intervention dosage, non-CP services; Morgan-López et al., 2026; Saavedra et al., 2023). However, these factors would add complexity to an already complex set of models, and a number of these factors are part of planned analyses beyond the current study (Morgan-López et al., 2022).

An additional limitation stems from the unbalanced sample of studies available to the current IDA with respect to age level. Conclusions with respect to main and moderator effects of treatments are clearest when each of the treatment conditions is implemented at each level (elementary, middle, high school) in a balanced factorial design. In the present study, MCP, IECP, and ICP were implemented only in elementary school samples. Ideally, future studies will be conducted that include multiple implementations of each variant of CP at each of the levels to provide a more balanced data set for a future IDA. Special attention should be given to implementing MCP and IECP in middle and high school, particularly given known developmental differences in the strength of the links between age of onset for STBs and risk for later suicidal behaviors (Whalen et al., 2015). Further, indirect effects were detected through teacher-reported intermediate endpoints, whereas parent-report outcomes showed total effects favoring CP but no mediation (McDaniel et al., 2025). This discrepancy highlights potential discrepancies in informant perspectives and suggests that additional work is needed to understand how different sources of informant information contribute to intervention effect estimates. It is possible that the intermediate endpoints for which there were no mediation effects (e.g., somatization) might have been better captured via child self-report (see Funder & West, 1993 for a discussion).

### Future Research

Several avenues for future research emerge from these findings. First, a critical next step is to examine mechanisms of action (MoAs) versus intermediate endpoints. Although the present study focused on indirect effects through intermediate psychiatric endpoints, further investigation is needed to understand how CP interventions influence STBs through intervention-specific mechanisms, such as improvements in self-regulation, coping skills, or social connectedness. Second, given the observed biological sex-specific pathways where boys benefited more from CP through reductions in externalizing problems (e.g., aggression, conduct problems) and girls showed some evidence of effects through internalizing pathways (i.e., anxiety), future studies of STB crossover effects studies should explicitly test differential intervention mechanisms by biological sex. This work could inform biological sex-tailored/enhanced adaptations of CP.

## Conclusion

Prevention science is based on a principle that interventions should address fundamental causal processes to affect later outcomes (Coie et al., 1993; Conduct Problems Prevention Research Group, 2020). This study tested that principle by exploring previously unexamined indirect effects on later suicidal thoughts and behaviors of a Coping Power intervention which is proximally focused on reduction of aggression and conduct problems. Although Coping Power was developed without consideration of suicidal thoughts and behaviors as a future outcome, the fact that the intervention addresses mechanisms that are common for both externalizing outcomes and for suicidal thoughts and behaviors, and that aggressive behaviors are themselves risk factors for STB, provided the foundation for this study. Notably, it was found that the CP intervention produced reductions in aggression and conduct problems (and in other proximal outcomes related to hyperactivity and internalizing problems), that, in turn, led to reduced risk for STB for these at-risk youth. Because the study capitalizes on the unique benefits of an IDA approach to compare different adapted variants of CP, the study provided insight into the relative utility and mechanisms of operation of the different CP versions in addressing STB. It also identified how youths’ biological sex and developmental level moderated these effects. In this way, the study provides an impetus for further research on the tailoring of CP and related cognitive behavioral interventions to prevent future STB.

## Supplementary Information

Below is the link to the electronic supplementary material.

ESM 1 (XLSX 28.4 KB)

ESM 2 (TXT 2.59 KB)

## Data Availability

Deidentified, individual-level participant data will be available via the National Institute of Mental Health Data Archive at the conclusion of the funded project. Mplus code for the analyses presented are available in the Supplemental Materials.

## Footnotes

1 Saavedra et al. (2023) noted that there is generally insufficient variability among low base rate items for estimation of factor loading DIF.

2 An asterisk (*) next to the reported *p* value denote effects that did not contain 0 in the bias-corrected bootstrap 2-tailed 95% confidence interval that had a parametric *p* value greater than.05. Effects with a double-asterisk (**) denote effects where 0 is not in the bias-corrected bootstrap confidence interval for the individual path coefficient but the corresponding indirect effect does not contain 0 in the confidence interval.
